# Has the Step-Up Approach Improved Prognosis in Severe Necrotizing Acute Pancreatitis?

**DOI:** 10.3390/jcm15082881

**Published:** 2026-04-10

**Authors:** Ricardo Gadea-Mateo, Marina Garcés-Albir, Dimitri Dorcaratto, Georgy Kadzhaya-Khlystov, Vicente Sanchiz, Elena Muñoz-Forner, Rosana Villagrasa, Isabel Mora-Oliver, Elisabetta Casula, Mar Juan-Diaz, Pablo Navarro-Cortés, Jorge Guijarro-Rosaleny, Isabel Pascual-Moreno, Luis Sabater

**Affiliations:** 1 Department of Surgery, Hospital Clínico Universitario of Valencia, Biomedical Research Institute INCLIVA, University of Valencia, 46010 Valencia, Spain; rgadeamateo@gmail.com (R.G.-M.); georgykadzhaya@gmail.com (G.K.-K.); 2 Department of Surgery, Liver-Biliary and Pancreatic Unit, Hospital Clínico Universitario of Valencia, Biomedical Research Institute INCLIVA, University of Valencia, 46010 Valencia, Spain; dorcaratto.dimitri@gmail.com (D.D.); emufor@hotmail.com (E.M.-F.); isab.mora11@gmail.com (I.M.-O.); luis_sabater@hotmail.com (L.S.); 3 Department of Gastroenterology, Hospital Clínico Universitario of Valencia, 46010 Valencia, Spain; vsanchizs@hotmail.com (V.S.); r_villagrasa@yahoo.es (R.V.); pnavarrocortes@gmail.com (P.N.-C.); isabel.pascual-moreno@uv.es (I.P.-M.); 4 Department of Radiology, Hospital Clínico Universitario of Valencia, 46010 Valencia, Spain; elisabettacas@gmail.com (E.C.); jorguijarro@gmail.com (J.G.-R.); 5 Critical Care Unit, Hospital Clínico Universitario of Valencia, 46010 Valencia, Spain; mar604@hotmail.com

**Keywords:** acute pancreatitis, Video-Assisted Retroperitoneal Debridement (VARD), step-up approach

## Abstract

**Background/Objectives**: Acute pancreatitis is a prevalent pathology with increasing incidence. Despite advances in treatment, some patients still present a severe clinical course with high morbidity and mortality rates. We evaluated the association between implementation of a step-up-based management strategy and clinical outcomes in patients with severe acute pancreatitis (SAP) treated at a tertiary referral center. **Method**: A retrospective observational study was conducted, including patients treated for SAP at a tertiary care center. Clinical outcomes, including mortality, morbidity, and length of hospital stay, were compared between two periods: Period A (1998–2010, classical treatment) and Period B (2011–2021, step-up approach). A subanalysis on minimally invasive techniques was also performed for Period B. **Results**: In total, 116 patients were included (39 Period A; 77 Period B). Pancreatic fistulas were reduced in Period B (15.38% vs. 5.33%; *p* = 0.088), as was mortality (30.76% vs. 18.67%; *p* = 0.15). Open surgeries decreased significantly in Period B (71.9% vs. 16.9%; *p* = 0.043), as did the mean hospital stay (60.5 ± 28 vs. 33.08 ± 28 days; *p* < 0.001). When comparing endoscopy management versus Video-Assisted Retroperitoneal Debridement (VARD), the rate of pancreatic fistulas was higher in the VARD group (0% vs. 57.1%; *p* < 0.01). Patients requiring VARD presented with larger collections (710 cc vs. 1737.9 cc; *p* = 0.03) and fewer procedures (4.2 ± 2.3 vs. 1.5 ± 0.5; *p* = 0.002). **Conclusions**: The step-up management in patients with SAP was associated with a decrease in open surgical approches and length of stay. VARD was performed in patients with higher volume collections and was associated with fewer interventions than patients treated by endoscopic necrosectomy; however, the incidence of pancreatic fistulas was higher.

## 1. Introduction

Acute pancreatitis (AP) is the third most frequent gastrointestinal disorder in the United States, with an estimated annual incidence in Spain of 35–40 cases per 100,000 inhabitants [1]. It causes 275,000 hospitalizations per year, representing a significant health expenditure and resource utilization within healthcare systems [2]. Its incidence is currently increasing, probably due to rising obesity rates and metabolic syndrome prevalence worldwide [3]. Gallstones and alcohol consumption remain the most common etiologies, although hypertriglyceridemia and post-procedural pancreatitis are increasingly observed in clinical practice.

Approximately 20% of patients with severe acute pancreatitis (SAP) develop necrosis of the pancreatic and peripancreatic tissues [4,5]. Pancreatic necrosis is a serious complication that compromises the prognosis of the disease and is associated with a more aggressive inflammatory response [6]. On many occasions, these patients will develop critical complications, such as multiorgan failure, systemic inflammatory response syndrome, infection of the necrosis leading to prolonged intensive care admission, or even death [7,8]. The mortality rates in SAP are around 17–30% [9,10] and are significantly higher when infected necrosis is present. Early identification of patients at risk of deterioration and timely intervention are therefore essential to improve outcomes.

Current management of SAP with infected necrosis is structured according to the “step-up approach”, which prioritizes less invasive measures before escalating to more aggressive treatments [7]. This strategy aims to reduce the surgical trauma and procedure-related complications. The use of minimally invasive necrosectomy has increased in recent years [3,6,8,11,12]. Even so, the mortality rate after pancreatic necrosectomy in specialized centers can still reach 25% [7,10,13], highlighting the complexity of these patients and the need for continuous refinement of treatment protocols.

This study aimed to analyze whether the implementation of the step-up approach improved outcomes (morbidity, mortality and length of stay) in patients with SAP in our center. Additionally, we sought to compare the clinical results among those patients requiring endoscopic necrosectomy or VARD, in order to better define the role of each therapeutic strategy within the current management algorithm.

## 2. Material and Methods

This is a retrospective observational study including patients treated for SAP at the Hospital Clínico Universitario of Valencia (HCUV) from January 2011 to December 2021. These patients were compared with a previously published cohort of SAP patients treated between 1998 and 2010 [14]. The step-up approach was officially implemented at our center in 2011 for the management of infected necrotizing pancreatitis, representing a major institutional change in therapeutic strategy.

The study was conducted and reported in accordance with the STROBE (Strengthening the Reporting of Observational Studies in Epidemiology) guidelines for retrospective observational studies, ensuring transparency in patient selection, data collection, variable definition and outcome reporting.

Diagnosis of AP was based on compatible clinical presentation, elevated serum amylase levels (>3 times the upper normal limit) and/or suggestive radiological findings. All patients were managed according to the hospital protocol, which included intensive fluid therapy during the first 48 h of admission, adequate analgesia, and prophylactic low-molecular-weight heparin. Early enteral nutrition was encouraged whenever feasible, preferably via oral intake or enteral nutrition through a nasojejunal tube. Re-evaluation with contrast-enhanced computed tomography (CT) was performed when there was clinical deterioration, suspected complications, or lack of improvement. The volume of pancreatic or peripancreatic collections was calculated by measuring the maximal axial, coronal, and sagittal diameters on the initial CT scan obtained prior to the first procedure, and the volumetric estimation was expressed in cubic centimeters.

Patient selection was performed through the hospital documentation service by filtering electronic medical records using the diagnosis code for acute pancreatitis. The inclusion criteria were age ≥ 18 years, admission for severe acute pancreatitis according to the revised Atlanta criteria [15] and the availability of complete clinical and radiological data. The exclusion criteria included incomplete medical reports, chronic pancreatitis, and patients transferred after definitive intervention elsewhere.

SAP was defined by the presence of persistent organ failure (>48 h), with or without local complications.

Infected necrosis was defined as the presence of gas within pancreatic or peripancreatic collections on contrast-enhanced CT and/or positive microbiological culture obtained from percutaneous, endoscopic, or surgical samples, together with compatible clinical signs of infection. In selected cases, infection was presumed based on persistent sepsis despite optimal supportive treatment and lack of clinical improvement [16].

A pancreatic fistula was defined as the persistent drainage of amylase-rich fluid and/or imaging evidence of pancreatic duct disruption requiring ongoing drainage or intervention. Clinically relevant fistulas were categorized according to their clinical impact, acknowledging that the International Group for Pancreatic Surgery (ISGPS) criteria were originally developed for postoperative settings [17].

Treatment decisions during both periods were made within a multidisciplinary team including a gastroenterologist, interventional radiologists, general surgeons, and critical care physicians, ensuring a standardized and consensus-based approach.

Until 2010, surgical treatment for infected necrosis primarily involved open necrosectomy and lavage drains according to the Beger or Bradley technique [18,19], with repeated reinterventions as necessary. In Period B, a step-up approach was implemented [14]. Initial management included initial percutaneous and/or transgastric drains, followed by endoscopic necrosectomy when required. Escalation to VARD was considered in patients with inadequate response after endoscopic and/or percutaneous drainage, defined by persistent sepsis, lack of clinical improvement, persistent or worsening organ failure and/or persistent large residual necrotic collections on follow-up imaging considered not amenable to further effective endoscopic treatment [20]. Open surgery was reserved for exceptional circumstances such as bowel perforation, abdominal compartment syndrome, or uncontrolled bleeding.

The Ranson Criteria, Acute Physiology and Chronic Health Evaluation II (APACHE II) score at admission, and C-reactive protein (CRP) level at 48 h were used to assess the severity of pancreatitis. The body mass index (BMI) was measured, and comorbidities were calculated based on the Charlson Comorbidity Index (CCI) [21]. Organ failure was defined according to the revised Atlanta classification [15]. The duration of organ failure was considered persistent if it was maintained for >48 h, and multiple organ failure (MOF) was defined as the presence of two or more organ failures derived from admission.

Data were obtained from medical records and anonymized in a database. The study was approved by the Clinical Research Ethics Committee of the HCUV.

Continuous variables were expressed as the means with standard deviations or the medians with ranges when appropriate. Categorical variables were summarized as frequencies and percentages. Comparisons between groups were performed using the Chi-square (X^2^) test or Fisher’s exact probability test for categorical variables, and the two-tailed Mann–Whitney U test for continuous variables. A *p*-value < 0.05 was considered statistically significant.

Artificial intelligence (AI) tools were used exclusively for English language editing and grammar improvement. No AI tools were used for data analysis or scientific content generation. The authors reviewed and approved the final manuscript and take full responsibility for its content.

## 3. Results

**Figure 1:**
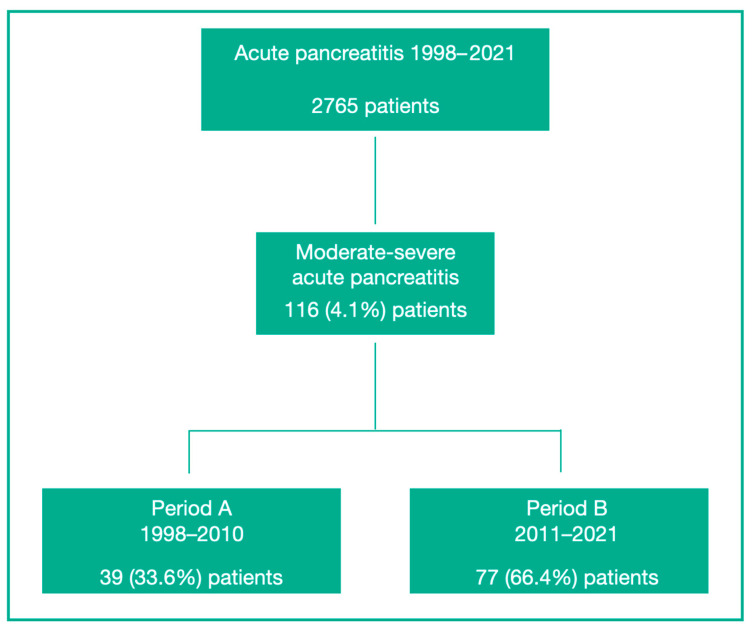
Flowchart of study patients.

Across both study periods, 2765 patients were diagnosed with AP. During Period B, 1388 patients presented with AP, and 77 patients (5.5%) met the SAP criteria. These were compared with 39 patients with SAP treated in Period A (1998–2010) (Figure 1).

The baseline demographics and clinical characteristics at admission were similar between groups (Table 1); however, the severity markers were discordant. The APACHE II scores were significantly higher in Period B, whereas Ranson scores were higher in Period A. The CRP levels at 48 h after admission were significantly higher in Period B patients (371.5 vs. 228.65 mg/L; *p* < 0.01).

The morbidity, mortality and hospital stay from the onset of the episode in both study periods are shown in Table 2. The mortality decreased from 30.76% in Period A to 18.67% in Period B, although this did not reach statistical significance (*p* = 0.15). The rates of persistent organ failure (>48 h), multiorgan failure, respiratory failure, shock and ICU admission were similar between groups.

Open surgeries were significantly reduced after implementation of the step-up protocol (71.9% vs. 16.9% patients; *p* = 0.043). The mean hospital stay decreased significantly from 60.5 ± 28 in Period A to 33.08 ± 28 days in Period B (*p* < 0.001). When readmissions within 90 days were included, the hospital stay remained significantly shorter in Period B (*p* < 0.01).

Pancreatic fistulas (ISGPS grade B/C) decreased from 15.38% to 5.33% (*p* = 0.088), and intra-abdominal bleeding increased from 15.38% to 20% (*p* = 0.57).

**Figure 2:**
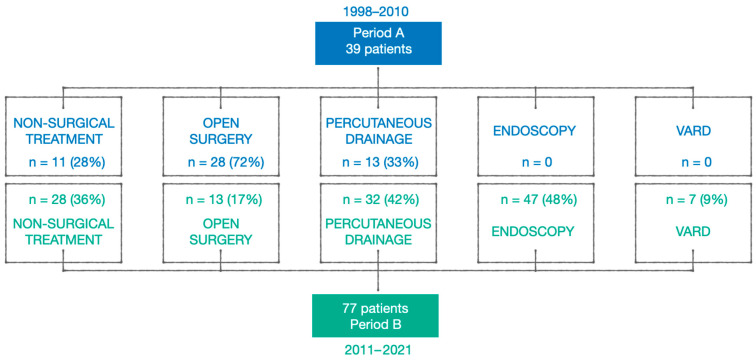
Distribution of patients from Period A (*n* = 39) and Period B (*n* = 77) based on the type of treatment.

Figure 2 illustrates the distribution of treatment modalities in both periods, demonstrating a marked reduction in open surgical approaches and an increase in minimally invasive strategies during Period B.

**Figure 3:**
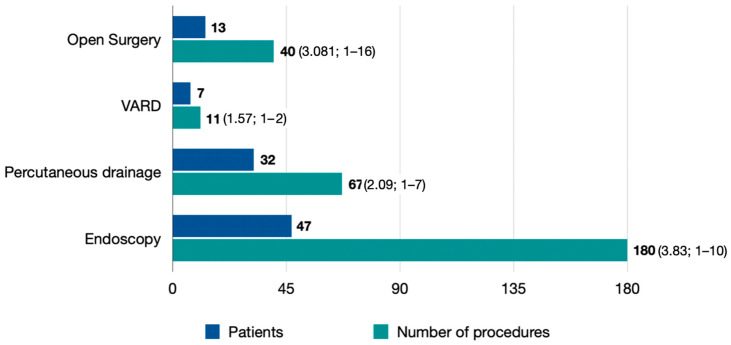
Number and type of procedures performed per patient during Period B.

In Period B, 180 endoscopic procedures were performed in 47 patients. Of these, 37 patients underwent endoscopic necrosectomy. The total number of procedures was 157 in the endoscopy group and 11 in the VARD group (Table 3). The mean number of procedures per patient was significantly higher in the endoscopy group (4.2 ± 2.3 vs. 1.5 ± 0.5; *p* = 0.002) (Figure 3).

Comparison between the endoscopic necrosectomy patients and VARD patients in Period B (Table 3) showed no significant differences in age, APACHE II, Ranson score, or organ failure rates. However, the VARD patients had a larger collection size at admission (710 cc vs. 1737.9 cc; *p* = 0.03), and a collection size reduction higher than 50% after intervention was more frequently achieved in the VARD group (88.9% vs. 25.4%; *p* < 0.01).

Pancreatic fistula occurred in four VARD patients (57.1%) and in none of the endoscopy patients (*p* < 0.01). All fistulas were managed conservatively.

The post-procedural hemorrhage and pseudocyst formation were similar between groups. The mortality was 6.4% in the endoscopy group and 14.3% in the VARD group (*p* = 0.6). The hospital stay after the first procedure was shorter in the VARD group (34.2 vs. 60.9 days; *p* = 0.04), although the total stay did not differ significantly.

## 4. Discussion

A notable increase in the rate of SAP cases over time was highlighted in this study, rising from 33.6% in Period A to 66.4% in Period B [22]. This increase may reflect better referral patterns to tertiary centers, improved diagnostic capabilities, and a rising prevalence of metabolic risk factors. The centralization of complex pancreatic disease may also have contributed to the higher proportion of severe cases in the later period.

In our center, the current management of SAP follows the step-up approach, which was associated with a clinically relevant reduction in morbidity and mortality compared to open surgery (30.76 vs. 18.67%). These results are clinically meaningful, particularly considering the severity of the disease. Similar trends have been reported in other institutional series and meta-analyses, supporting minimally invasive strategies as the standard of care [6,7,8,11,23].

The divergent behavior of APACHE II and Ranson scores between periods suggests heterogeneity in baseline severity assessment, making it difficult to attribute a clear difference in overall disease severity to either cohort. Although statistical significance was not achieved for mortality reduction, the magnitude of decrease suggests a potential beneficial effect that may become significant in larger multicenter cohorts. The study is likely underpowered to detect differences in mortality. The variability between studies could be explained by the disparity in clinical cases and diverse treatment strategies employed by different hospitals [24]. Following implementation of the step-up approach, a reduction in open surgeries and pancreatic fistulas was observed. The progressive reduction in open surgery rates likely reflects improved patient selection and the effectiveness of percutaneous and endoscopic techniques in controlling sepsis and reducing inflammatory burden, as well as broader improvements in minimally invasive techniques, interventional radiology availability and multidisciplinary management over time [25]. Furthermore, the mean hospital stay was significantly reduced in Period B, with a reduction of nearly four weeks compared to the previous period. From a healthcare system perspective, this decrease in hospitalization days is particularly relevant, as patients with SAP frequently require prolonged admissions, repeated imaging, and multiple interventions, generating a considerable burden on intensive care and surgical resources.

Although the rate of intra-abdominal bleeding increased in Period B (15% to 20%; *p* = 0.57), this may be attributable to the higher number of endoscopies performed and the more frequent manipulation of necrotic cavities. These results are similar to previous publications on endoscopy in patients with acute pancreatitis [26,27,28]. Despite this, all patients with hemorrhage responded to conservative management with endoscopic intervention or angioembolization, and no procedure-related mortality was observed, supporting the overall safety of minimally invasive strategies when performed in specialized centers.

However, interpretation of these results requires caution, as the comparison spans more than two decades, during which multiple aspects of care evolved, including ICU management, nutritional support, imaging techniques, interventional radiology availability, and endoscopic expertise.

Endoscopy is now the first therapeutic step for symptomatic collections or pancreatic necrosis unresponsive to conservative measures [20,29]. However, the role of VARD remains less clearly defined, and the superiority of endoscopic approaches has not been definitively established [10,30]. Randomized trials are challenging due to the clinical heterogeneity of these patients and the variability in the anatomical extension of necrosis. In our study, VARD was associated with higher pancreatic fistula rates, which is inherent to this type of treatment and in accordance with previous studies [10,31,32,33]. All pancreatic fistulas responded to a non-surgical approach without extending the hospital stay, and no enteric fistulas were observed. This suggests that although VARD may carry a higher local complication rate, these events can be adequately controlled within a structured multidisciplinary setting. These findings should be interpreted with caution due to the small sample size in this analysis and be considered hypothesis-generating.

In our series, initial collection volumes (axial–coronal–sagittal axes) were larger in the VARD group (710 cc vs. 1737.9 cc; *p* = 0.03), suggesting that larger or anatomically complex collections are more likely to require surgical debridement. This observation supports the concept that the collection size and extension may be key determinants in selecting the optimal therapeutic strategy. Fewer procedures were needed for definitive treatment in the VARD group (mean 1.5 ± 0.5 vs. 4.2 ± 2.3; *p* = 0.002), in line with the published literature [23]. Moreover, a higher proportion of VARD patients achieved a reduction of more than 50% of the collection volume after intervention, indicating a more substantial immediate mechanical effect. This exploratory comparison should be interpreted with caution, as patients undergoing VARD presented with larger and more complex collections.

The number of procedures required for resolution in our study is slightly higher than reported in other studies, likely due to the exclusion from the analysis of patients who did not require endoscopic or surgical intervention to solve the episode [10,26].

All patients were treated following a treatment protocol according to the step-up approach. Open surgery was considered the most aggressive option and was reserved for exceptional cases such as intestinal perforation or abdominal compartmental syndrome. VARD was offered only in case of endoscopic and/or percutaneous drainage failure. This may explain the tendency toward a longer total hospital stay observed in VARD patients in our series (63.5 vs. 89.5 days; *p* = 0.08), as these patients likely represented more complex cases requiring escalation of therapy. However, the length of stay from the first necrosectomy (VARD or endoscopy) was shorter in surgical patients (60.9 vs. 39.2 days; *p* = 0.04), in accordance with the previous studies [23]. This finding suggests that once the decision for surgical debridement is made, resolution may occur more rapidly [34].

Indications for each type of approach remain to be clearly defined, though the collection size and location may help guide decision-making [35,36]. In recent randomized trials comparing endoscopy and surgery, patients were randomized into surgical or endoscopic groups, which may not offer the best scheme for each patient and may not be a fair comparison. In fact, they showed similar results in terms of total hospital stay and number of pancreatic fistula (lower in the endoscopy group) without reducing major complications or death [10,29]. Although ongoing studies aim to demonstrate the superiority of the endoscopic approach, the optimal selection criteria for patients who may benefit from early VARD remains undefined. In this context, our data suggest that VARD should be considered a complementary tool rather than a competing strategy, particularly in patients with large-volume necrosis or insufficient response to endoscopic therapy. However, endoscopy should still be considered the first-line intervention whenever feasible.

The main strength of this study is that it provides real-world clinically applicable insights into treatment selection within the step-up approach, based on a long-term experience in a tertiary referral center. It highlights that VARD may achieve comparable outcomes despite being preferentially reserved for more complex cases.

This study is limited by its retrospective nature, potential data biases, and modest sample size, particularly in subgroup analyses. The relatively small number of patients undergoing VARD limits the strength of comparative conclusions. Despite these limitations, the findings provide valuable insights that could be useful for designing future prospective and multicenter studies. The consistency of the trends observed with previously published series reinforces the external validity of our results. Based on the results of this study, we are conducting a national multicenter retrospective study (PROTEV) to determine the predictive factors of endoscopic treatment failure and, thus, better individualize therapeutic strategies in severe necrotizing pancreatitis.

Based on the results of our analysis, future studies may further explore potential predictors of endoscopic treatment failure, as well as better define its indications and optimal timing for VARD in patients with severe acute pancreatitis.

## 5. Conclusions

In conclusion, the implementation of the step-up approach was associated with improved clinical outcomes in SAP in our center, with a statistically significant decrease in open surgery approach and length of stay, and a clinically relevant but not statistically significant reduction in mortality from 30.76% to 18.67%. VARD resulted in a larger reduction in necrotic collections and required fewer interventions than endoscopy management, although it carried a higher risk of pancreatic fistulas. Larger multicenter studies are needed to better define optimal patient selection and treatment pathways.

## Figures and Tables

**Table 1:** Demographic and clinical data on admission.

	Period A (1998–2010) (*n* = 39)	Period B (2011–2021) (*n* = 77)	*p* Value
Age (mean ± SD)	64.55 (±15.6)	63.70 (±13.46)	0.22
Sex (male/female)	19/20	52/25	0.07
Etiology			0.6
Gallstones	25 (64.1%)	42 (57.5%)	
Alcohol	5 (12.8%)	15 (20.5%)	
Other	9 (23.1%)	16 (21.9%)	
BMI > 30	13/37 (35.13%)	15/47 (31.9%)	0.8
Comorbidity	22 (56.4%)	43 (58.9%)	0.8
CCI			0.8
1–2	16 (41%)	30 (41.1%)	
>2	6 (15.4%)	13 (17.8%)	
Ranson (mean ± SD)	4.7 (±2.0)	3.39 (±2.0)	<0.001
APACHE II (mean ± SD)	11.25 (±2.0)	16.51 (±6.66)	<0.001
CRP 48 h (mean ± SD)	228.65 (±132)	371.5 (±150.22)	<0.01

BMI: body mass index (Kg/m^2^); CCI: Comorbidity Charlson index; CRP: C reactive protein (mgr/L).

**Table 2:** Morbidity, mortality and hospital stay.

	Period A (1998–2010) (*n* = 39)	Period B (2011–2021) (*n* = 77)	*p* Value
Mortality	12 (30.76%)	14 (18.67%)	0.15
Organic failure > 48 h	5 (12.8%)	12 (16%)	0.86
MOF	14 (35.9%)	32 (42.7%)	0.61
Respiratory failure	18 (46.15%)	31 (41.33%)	0.8
Shock	15 (38.46%)	26 (34.67%)	0.08
Intraabdominal bleeding	6 (15.38%)	15 (20%)	0.57
Pancreatic fistula, grade B/C *	6 (15.38%)	4 (5.33%)	0.088
Open surgery	28 (71.79%)	13 (16.9%)	0.043
ICU stay	23 (59%)	37 (46.25%)	0.43
ICU stay (mean ± SD, range days)	23.9 ± 17.96 (1–80)	20.91 ± 23.31 (1–120)	0.5
Hospital stay (mean ± SD, range days)	60.05 ± 28.7 (4–129)	33.08 ± 28.12 (4–130)	<0.001
Hospital stay with readmission (mean ± SD, range days) **	60.05 ± 28.7 (4–129)	45.52 ± 32.27 (2–145)	<0.01

MOF: multiorgan failure; ICU: intensive care unit. * According to ISGPS classification [2]. ** Initial hospital stays plus readmission in the first 90 days.

**Table 3:** Demographic and clinical data and outcomes: endoscopy group vs. VARD group.

	ENDOSCOPY (*n* = 37)	VARD (*n* = 7)	*p* Value
Age (mean ± SD)	65.5 ± 12.3	57 ± 11.2	0.1
Sex (male/female)	21/16	4/3	0.98
CCI			<0.05
1–2	10 (27%)	5 (71.4%)	
>2	27 (73%)	2 (28.6%)	
Ranson (mean ± SD)	3.64 ± 1.8	3.67 ± 1	0.76
APACHE II (mean ± SD)	17.2 ± 7.8	14.7 ± 2.9	0.96
Shock	12 (32.4%)	3 (42.8%)	0.7
Renal failure	17 (45.9%)	2 (28.6%)	0.45
Respiratory failure	15 (40.5%)	3 (42.8%)	0.82
MOF	14 (37.8%)	3 (42.8%)	0.9
Organic failure			0.5
No	14 (37.8%)	4 (57.1%)	
Transient	7 (18.9%)	0 (0%)	
Persistent	16 (43.2%)	3 (42.8%)	
Mortality	3 (6.4%)	1 (14.3%)	0.6
Hospital stay (mean ± SD, range days)	63.5 ± 55.35 (13–268)	89.5 ± 14.7 (70–115)	0.08
Hospital stay from 1st procedure (mean ± SD, range days)	60.9 ± 80.6 (7–136)	34.2 ± 25.9 (16–82)	0.04
Total number of procedures	157	11	<0.01
Mean number of procedures required (mean ± SD)	4.2 ± 2.3	1.5 ± 0.5	0.002
Collection size (mean ± SD)	710 cc ± 754.4	1737.9 cc ± 1079.5	0.03
Collection size reduction >50% after procedure	15 (25.4%)	8 (88.9%)	<0.01
Drainage after procedure	10 (27%)	1 (14.3%)	0.83
Complications after procedure			
Hemorrhage	2 (5.6%)	0 (0%)	0.9
Fistula	0 (0%)	4 (57.1%)	<0.01
Pseudocyst	7 (14.9%)	1 (14.3%)	0.85

CCI: Comorbidity Charlson Index. MOF: Multiorgan failure.

## Data Availability

The original contributions presented in this study are included in the article. Further inquiries can be directed to the corresponding author.
